# Internet-Based Health Care Communication Among Cancer Survivors, 2011–2018 National Health Interview Survey

**DOI:** 10.5888/pcd18.210163

**Published:** 2021-09-09

**Authors:** Bian Liu, K. Robin Yabroff, Zhiyuan Zheng, Ronald Tamler, Xuesong Han

**Affiliations:** 1 Department of Population Health Science and Policy, Icahn School of Medicine at Mount Sinai, New York, New York; 2 Surveillance and Health Equity Science, American Cancer Society, Atlanta, Georgia; 3 Department of Endocrinology, Diabetes and Bone Disease, Icahn School of Medicine at Mount Sinai, New York, New York

## Abstract

**Introduction:**

Understanding trends and associated factors in internet-based health care communication (IBHC) among cancer survivors is important for meeting patient needs because their reliance on telehealth is growing. We aimed to examine IBHC use among cancer survivors in the US.

**Methods:**

We identified adult cancer survivors aged 18 to 64 (n = 8,029) and 65 or older (n = 11,087) from the National Health Interview Survey in 2011–2018. We calculated temporal trends of self-reported IBHC in the previous year (filled a prescription, scheduled a medical appointment, or communicated with a health care provider) and used multivariable logistic models to identify associated factors.

**Results:**

Approximately 84% of survivors had been diagnosed 2 years or more before the survey. IBHC prevalence increased among cancer survivors aged 18 to 64, from 19.3% to 40.2%, and among those aged 65 or older, from 11.4% to 22.6%, from 2011 to 2018 (*P* for trend <.001). Among both age groups, lower educational attainment, lack of usual source of care, and current smoking were associated with less IBHC, whereas residing in the South or West, having 1 or more chronic conditions, and drinking any alcohol were associated with higher IBHC (all *P* < .05). Factors associated with less IBHC also included being non-Hispanic Black or Hispanic, lacking private insurance, and being 11 or more years postdiagnosis among survivors aged 18 to 64; among survivors aged 65 or older, factors were being an older age, not married, and non-US born (all *P* < .05).

**Conclusion:**

IBHC among cancer survivors is common and increasing, with differences across sociodemographic and behavioral characteristics. As health care delivery continues adopting IBHC and other advanced telehealth techniques, disparities need to be addressed to ensure equitable access to care for all cancer survivors.

**Summary**

**What is already known about this topic?**

As a basic component of telehealth, internet-based health care communication (IBHC) establishes online patient–provider communication relationships and may facilitate adoption of advanced telehealth techniques.

**What is added by this report?**

In 2011–2018, self-reported IBHC increased among cancer survivors aged 18 to 64 years (from 19.3% to 40.2%) and 65 or older (from 11.4% to 22.6%). Lower educational attainment and lack of usual source of care were associated with lower IBHC in both age groups. Furthermore, minority race/ethnicity and lack of private insurance were associated with lower levels of IBHC use among younger survivors.

**What are the implications for public health practice?**

The identified IBHC patterns can help address disparities in IBHC and other telehealth services use for cancer survivors.

## Introduction

Telehealth encompasses the use of electronic information and telecommunication technologies to support and promote care delivery, health-related education, and public health (1,2). During 2020, health care shifted rapidly toward telehealth and telemedicine in response to the COVID-19 pandemic (3). In December 2020, the Centers for Medicare & Medicaid Services announced that 60 of the 144 newly added telehealth services during the pandemic will become permanent (4). Supported by payment policies and maturing innovative communication technology, such as wide adoption of electronic health record systems and video conferencing techniques, telehealth will likely continue to grow (2–5).

As telehealth expands, it is important to examine the use of its basic components, such as internet-based health care communication (IBHC), before the COVID-19 pandemic. Established online communication systems and relationships between patients and providers can facilitate effective adoption of more advanced telehealth techniques such as patient care portals, video visits, and remote patient monitoring. For example, although patient portals have become ubiquitous (6), some patients face obstacles in using them. A recent study based on a national survey collected during 2014–2018 showed that patients who had used IBHC were more likely than patients who had not used IBHC to use patient portals (7). Therefore, understanding the trends and factors associated with IBHC will help in evaluating the capacity of wider adoption of more sophisticated telehealth technologies during and beyond the pandemic. Moreover, it is essential to identify populations with barriers to IBHC so that the advancement of telehealth, which is intended to increase access and improve care delivery, will not perpetuate existing health disparities (2,8,9).

Telehealth has been used in the delivery of cancer survivorship care in health and health behavior promotion (eg, tobacco, alcohol, mental health evaluation and counseling) with demonstrated acceptability and feasibility (10–15). During the COVID-19 pandemic, the dependence on telehealth among this high-risk population of cancer survivors intensified (16). Understanding IBHC use among cancer survivors can inform the ongoing transition toward delivery of care through telehealth to cancer survivors. We postulate that cancer survivors may have unique needs and challenges in adopting IBHC, as a result in part of rapid declines in health, potentially lengthy and complicated treatment regimens, and lasting effects of treatment. Older studies found that cancer survivors were more likely than people without a cancer history to use the internet for health-related purposes (17) and use more online patient–provider communication (18). On the other hand, cancer survivors also face barriers in using IBHC. The median age of cancer diagnosis is 66 years in the US; most cancer survivors — approximately 64% — are older adults (19). Advancing age was identified as a risk factor of not using health information technology in previous studies (20–23). Declining cognitive and physical functioning combined with telecommunication devices and applications that may not be older-adult–friendly could also make using IBHC and learning new technology challenging for older cancer survivors (24). Other factors such as low education level, low health literacy, comorbid conditions, and inadequate internet and health care access may also limit IBHC use, and the associations may be age-dependent (9,17).

Studies on cancer survivors’ IBHC use based on nationally representative samples generally used data from a relatively short period or may not reflect current use (17,18,25). Additionally, earlier studies used a broad definition of health information technology that encompassed activities with and without interactions with health care providers, such as seeking health information online and participating in group chats (9,17,25). Other studies evaluated the use of health information technology among the general population or among people with chronic conditions, but not specifically among cancer survivors (21–23). An updated examination of long-term trends and a focus on IBHC use that includes direct interaction with providers among cancer survivors is needed. In addition, given the age distribution of cancer and health technology use (19,24), it is also important to study younger and older cancer survivors separately. To that end, this study assessed the prevalence of IBHC use and associated factors among younger and older cancer survivors in the US during the past decade. The study findings can inform continued population-level evaluations of IBHC use during and after the COVID-19 pandemic and guide identification of targeted populations for promoting equity in IBHC use among cancer survivors.

## Methods

We used the publicly available 2011–2018 National Health Interview Survey (NHIS) to identify cancer survivors (26). We chose the study period to include the most recent data on IBHC, when questions on IBHC were consistently surveyed. The NHIS is representative of the US noninstitutionalized civilian population. One adult per family from a household was randomly selected for the interview. The final sample adult response rate ranged from 53.0% to 66.3% (26). Because these de-identified data are in the public domain, the study did not require institutional review board approval.

Cancer survivors were identified from the question, “Have you EVER been told by a doctor or other health professional that you had . . . cancer or a malignancy of any kind?” They were subsequently asked about the cancer type. We identified 25,173 adults who reported a cancer history and IBHC information. After excluding 5,185 adults with only a nonmelanoma skin cancer history, which is a relatively common nonfatal cancer and often excluded from cancer surveillance (19), and 872 adults with missing information on covariates, we included 19,116 survivors in the analytic sample.

**Outcome measures.** We defined IBHC use as a positive answer to any of 3 questions: “DURING THE PAST 12 MONTHS, have you ever used computers for any of the following . . .” asking about 1) “Fill a prescription,” 2) “Schedule an appointment with a health care provider,” and 3) “Communicate with a health care provider by email.” We also examined each IBHC type separately.

**Exposure measures.** Informed by previous research (8,9,17,18,20–23,25), we included measures from 4 domains to examine factors associated with IBHC: sociodemographic characteristics, health care access, health status, and health behaviors. Sociodemographic characteristics were age group, sex, race/ethnicity, educational attainment, marital status, place of birth, and residence region. For health care access, we used data on health insurance type and whether a respondent had a usual source of care. For participants’ health status, we included years since cancer diagnosis, number of chronic conditions, psychosocial stress measured by the Kessler scale (27), and body mass index (BMI) categories. We also included self-reported health behavior measures on smoking and drinking alcohol.

### Statistical analysis

We stratified all analyses by age group (18–64 and ≥65) to reflect differences in health insurance coverage, cancer risk, and internet use. Among older adults (aged ≥65), we used 5-year age intervals, because cancer is more common among older adults (65–69, 70–74, 75–79, ≥80). In the younger age group, we used 10-year age intervals or longer (18–34, 35–44, 45–54, 55–64) to ensure a sufficient sample size in each age category. We assessed the temporal trend in IBHC by fitting univariable logistic regression models that treated survey year as a continuous variable. We assessed factors associated with IBHC use with multivariable logistic regression models. We included survey year as a covariate to account for the temporal variations during the study period. We reported the adjusted prevalence ratios (aPRs) and their 95% CIs.

All analyses accounted for the complex NHIS survey design by incorporating sampling strata, cluster, weight, and domain parameters into survey procedures for population-level variance estimation (26). We conducted analyses in SAS version 9.4 (SAS Institute Inc), SAS-callable SUDAAN (SAS Institute Inc), and R version 4.0.2 (R Core Team) with RStudio version 1.3.1073 (RStudio Team).

## Results

The 8,029 survivors aged 18 to 64 and 11,087 survivors aged 65 or older surveyed from 2011 through 2018 represented 7,343,694 and 8,270,633 weighted populations, respectively (Table 1). Most (83.9%) cancer survivors were diagnosed 2 or more years before the survey. Non-Hispanic Black and Hispanic survivors represented less than 10% of the sample in both age groups. Most survivors had at least a high school education. The proportion without a usual source of care when sick was 6.8% among cancer survivors aged 18 to 64 and 1.7% among cancer survivors aged 65 or older. Participants who currently smoke or drink alcohol represented 21.6% and 66.4% of cancer survivors aged 18 to 64, respectively, and 7.3% and 52.4%, respectively, among survivors aged 65 or older.

**Table 1:** Characteristics of Cancer Survivors Who Participated in the 2011–2018 National Health Interview Survey

Characteristic	Weighted %		
	Overall^a^	Aged 18–64	Aged ≥65
**All**	100.0	47.0	53.0
**Age, y**			
18–34	18.4	9.9	—
35–44	19.4	13.8	—
45–54	23.5	26.8	—
55–64	38.8	49.5	—
65–69	18.4	—	25.9
70–74	19.4	—	24.4
75–79	23.5	—	20.5
≥80	38.8	—	29.2
**Sex**			
Male	41.8	35.0	47.9
Female	58.2	65.0	52.1
**Race/ethnicity**			
Non-Hispanic White	81.6	78.9	83.9
Non-Hispanic Black	7.6	7.9	7.3
Hispanic	6.5	8.4	4.8
Other^b^	4.3	4.8	3.9
**Educational attainment**			
<High school diploma	12.7	9.9	15.2
High school diploma	27.2	25.0	29.3
>High school diploma	60.0	65.1	55.5
**Marital status**			
Married	58.5	59.8	57.3
Not married	41.5	40.2	42.7
**Place of birth**			
US born	90.7	90.5	90.9
Non-US born	9.3	9.5	9.1
**Residence region**			
Northeast	18.4	17.8	19.0
Midwest	23.9	24.4	23.5
South	36.9	36.7	37.0
West	20.8	21.1	20.5
**Insurance type**			
Private	58.8	67.3	—
Uninsured	18.2	23.3	—
Public only	21.4	9.5	—
Medicare + private	58.8	—	51.4
Medicare + public	18.2	—	13.7
Medicare only	21.4	—	32.0
Private and/or public without Medicare	1.6	—	2.9
**Have usual place to go when sick**			
Yes	95.9	93.2	98.3
No	4.1	6.8	1.7
**No. of chronic conditions^c^**			
0	23.3	34.3	13.5
1	25.2	27.7	22.9
2	20.8	17.4	23.8
≥3	30.7	20.6	39.7
**Psychological distress^d^**			
Kessler scores <13	95.0	92.6	97.3
Kessler scores ≥13	5.0	7.4	2.7
**Body mass index (BMI), kg/m^2^**			
Normal or underweight (<24.9)	32.1	31.1	33.0
Overweight (25.0 to <29.9)	34.1	31.3	36.6
Obese (≥30.0)	33.8	37.6	30.3
**Years since cancer diagnosis^e^**			
<2	16.1	17.4	15.0
2–5	26.3	28.4	24.4
6–10	20.8	20.5	21.1
≥11	36.8	33.7	39.6
**Smoking**			
Never	48.4	50.5	46.6
Former	37.5	27.9	46.1
Current	14.0	21.6	7.3
**Drinking**			
Lifetime abstainer	17.8	14.1	21.1
Former	23.2	19.5	26.5
Current (infrequent/light/unknown frequency)	40.9	47.3	35.2
Current (moderate/heavy)	18.1	19.1	17.2
**Survey year**			
2011	11.5	12.1	11.0
2012	11.5	12.1	11.1
2013	12.0	12.5	11.6
2014	11.8	12.2	11.4
2015	12.3	11.6	12.8
2016	13.5	13.4	13.6
2017	13.8	13.6	13.9
2018	13.6	12.6	14.5

Abbreviation: —, does not apply.

a The denominator for the overall column is all age groups combined. The unweighted study sample size for overall was 19,116; for aged 18–64 years, 8,029; and for aged ≥65 years, 11,087. The corresponding survey weighted sample size was 15,614,326; 7,343,694; and 8,270,633, respectively. Weighted percentage of the study sample overall was estimated by considering the complex NHIS survey design.

b Included non-Hispanic ethnicity with one of the following race groups: Native American/Alaska Native only, Asian only, race group not releasable, and multiple race.

c Chronic conditions considered were hypertension, high cholesterol, heart diseases (coronary heart disease, angina pectoris, heart attack, other heart condition or heart disease), stroke, emphysema, chronic obstructive pulmonary disease, asthma, diabetes, and arthritis.

d The Kessler Psychological Distress Scale (27) consists of 6 questions that ask about feelings of sadness, nervousness, restlessness, worthlessness, hopelessness, and feeling like everything is an effort during the past 30 days. Participants were asked to respond on a Likert scale ranging from none of the time (score = 0) to all of the time (score = 4); a cutoff of 13 was used to dichotomize psychological distress into serious (score ≥13) and not serious (<13).

e Years since cancer diagnosis was calculated by subtracting age at the interview and self-reported age at cancer diagnosis. Up to 3 cancer diagnoses were reported; for those who had 1 cancer (~10% of the sample), we used the shorter time between cancer diagnosis and survey interview.

Any IBHC use increased from 19.3% in 2011 to 40.2% in 2018 among cancer survivors aged 18 to 64 years, and from 11.4% to 22.6% among survivors aged 65 or older (both *P* for trend <.001) (Figure). We observed similar trends for each IBHC type among both age groups (all *P* ≤ .001). For both age groups, the magnitude of the increase was larger for using IBHC to schedule medical appointments (from 4.9% to 20.5% for survivors aged 18–64 and from 2.7% to 10.6% for survivors aged ≥65) or communicate with health care providers (from 8.9% to 28.1% for survivors aged 18–64 and from 5.5% to 15.4% for survivors aged ≥65) than for using IBHC to fill a prescription (from 13.1% to 18.5% for survivors aged 18–64 and from 7.3% to 11.7% for survivors aged ≥65). Any IBHC use also varied across the most common cancer types (data not shown).

**Figure:**
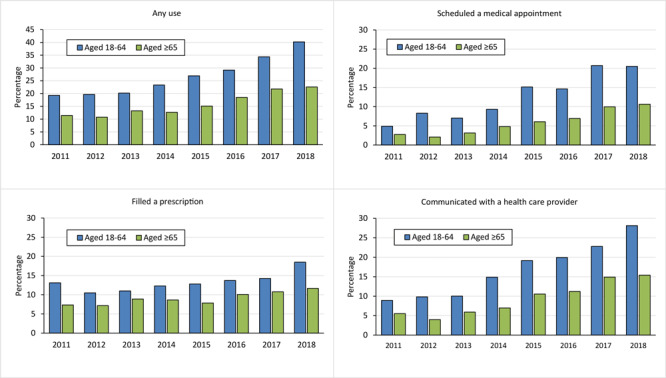
Increasing trend in internet-based health care communication use among cancer survivors stratified by age group, National Health Interview Survey 2011–2018. Any use of internet-based health care communication was defined as any use, in the past 12 months, of the following 3 types: communicated with a health care provider by email; filled a prescription on the internet; and scheduled a medical appointment on the internet. *P* value for the time trend was determined by using univariate logistic regression, where the dichotomized usage of internet-based health care communication (yes vs no) was the outcome variable, and survey year, treated as a continuous variable, was the explanatory variable. All *P*s for trend ≤ .001.

**Table:** 

Year	Aged 18-64	Aged ≥65
Any use		
2011	19.3	11.4
2012	19.7	10.7
2013	20.2	13.2
2014	23.3	12.7
2015	26.9	15.1
2016	29.2	18.5
2017	34.4	21.8
2018	40.2	22.6
Filled a prescription		
2011	13.1	7.3
2012	10.5	7.2
2013	11.0	8.9
2014	12.3	8.6
2015	12.8	7.8
2016	13.7	10.1
2017	14.2	10.8
2018	18.5	11.7
Scheduled a medical appointment		
2011	4.9	2.7
2012	8.3	2.1
2013	7.0	3.1
2014	9.3	4.8
2015	15.1	6.1
2016	14.6	6.9
2017	20.7	10.0
2018	20.5	10.6
Communicated with a health care provider		
2011	8.9	5.5
2012	9.8	4.0
2013	10.0	5.9
2014	14.9	7.0
2015	19.1	10.5
2016	19.9	11.2
2017	22.8	14.9
2018	28.1	15.4

Numerous factors significantly associated with any IBHC were largely similar between younger and older cancer survivors (Table 2). For example, lower use of IBHC was associated with educational attainment less than high school (aged 18–64: aPR = 0.39; 95% CI, 0.29–0.53; aged ≥65: aPR = 0.19; 95% CI, 0.12–0.29) or equivalent to high school (aged 18–64: aPR = 0.52; 95% CI, 0.45–0.61; aged ≥65: aPR = 0.46; 95% CI, 0.39–0.54), compared with education attainment above high school. Lower IBHC was also associated with lack of usual source of care (aged 18–64: aPR = 0.62; 95% CI, 0.47–0.81; aged ≥65: aPR = 0.52; 95% CI, 0.29–0.93), and current smoking (aged 18–64: aPR = 0.69; 95% CI, 0.60–0.80; aged ≥65: aPR = 0.60; 95% CI, 0.45–0.80), compared with never smokers. On the other hand, higher IBHC was significantly associated with living in the South or West, compared with the Northeast. Having 1 or more chronic conditions was associated with higher IBHC, compared with having no chronic conditions. In addition, compared with lifetime alcohol abstainers, participants who reported drinking alcohol had higher IBHC among younger (aPR range, 1.60–1.85; all 95% CIs >1) and older survivors (aPR range, 1.27–1.83; all 95% CIs >1).

**Table 2:** Factors Associated With Any Use of Internet-Based Health Care Communication^a^ Among Cancer Survivors, Stratified by Age Group, National Health Interview Survey 2011–2018

Characteristic	Adjusted Prevalence Ratio (95% CI)	
	Aged 18-64	Aged ≥65
**Demographic**		
**Age, y**		
18–34	1 [Reference]	—
35–45	1.04 (0.88–1.24)	—
46–55	0.93 (0.79–1.10)	—
56–64	0.84 (0.71–0.99)	—
65–69	—	1 [Reference]
70–74	—	0.78 (0.69–0.89)
75–79	—	0.63 (0.54–0.72)
≥80	—	0.36 (0.30–0.44)
**Sex**		
Male	0.91 (0.83–1.01)	1.05 (0.94–1.19)
Female	1 [Reference]	1 [Reference]
**Race/ethnicity^b^**		
Non-Hispanic Black	0.71 (0.58–0.87)	0.81 (0.63–1.04)
Non-Hispanic White	1 [Reference]	1 [Reference]
Hispanic	0.78 (0.63–0.96)	0.85 (0.60–1.20)
Other	1.05 (0.84–1.30)	1.16 (0.87–1.54)
**Education**		
<High school diploma	0.39 (0.29–0.53)	0.19 (0.12–0.29)
High school diploma	0.52 (0.45–0.61)	0.46 (0.39–0.54)
>High school diploma	1 [Reference]	1 [Reference]
**Marital status**		
Married	1 [Reference]	1 [Reference]
Not married	0.91 (0.83–1.00)	0.73 (0.65–0.81)
**Nativity**		
US born	1 [Reference]	1 [Reference]
Non–US born	0.90 (0.75–1.09)	0.70 (0.54–0.90)
**Region**		
Midwest	1.02 (0.87–1.21)	1.15 (0.96–1.38)
South	1.18 (1.01–1.37)	1.25 (1.05–1.49)
West	1.43 (1.23–1.67)	1.64 (1.37–1.96)
Northeast	1 [Reference]	1 [Reference]

**Health Care Access**		
**Health insurance^c^**		
Private insurance	1 [Reference]	—
Uninsured	0.63 (0.53–0.73)	—
Public insurance	0.64 (0.52–0.79)	—
Medicare + private insurance	—	1 [Reference]
Medicare + public insurance	—	0.85 (0.72–1.01)
Medicare only	—	0.91 (0.82–1.03)
**Has usual place to go when sick**		
Yes	1 [Reference]	1 [Reference]
No	0.62 (0.47–0.81)	0.52 (0.29–0.93)

**Health Status**		
**No. of chronic conditions**		
None	1 [Reference]	1 [Reference]
1	1.17 (1.04–1.32)	1.23 (1.02–1.49)
2	1.34 (1.18–1.52)	1.37 (1.13–1.66)
≥3	1.27 (1.10–1.47)	1.41 (1.17–1.70)
**Psychological distress^d^**		
Kessler score ≥13	0.96 (0.80–1.15)	0.80 (0.54–1.18)
Kessler score <13	1 [Reference]	1 [Reference]
**Body mass index (BMI), kg/m^2^**		
Normal or underweight (<24.9)	1 [Reference]	1 [Reference]
Overweight (25.0 to <29.9)	1.10 (0.98–1.23)	1.06 (0.93–1.21)
Obese (≥30.0)	1.01 (0.89–1.13)	1.00 (0.87–1.15)
**Time since cancer diagnosis, y**		
≤2	1 [Reference]	1 [Reference]
2–5	0.95 (0.84–1.08)	0.95 (0.81–1.11)
6–10	0.96 (0.84–1.09)	1.01 (0.85–1.20)
≥11	0.78 (0.69–0.88)	0.93 (0.79–1.09)

**Health Behavior**		
**Smoking status**		
Former	1.01 (0.92–1.12)	1.14 (1.02–1.28)
Current	0.69 (0.60–0.80)	0.60 (0.45–0.80)
Never	1 [Reference]	1 [Reference]
**Alcohol consumption**		
Former	1.60 (1.30–1.98)	1.27 (1.02–1.57)
Infrequent or light	1.77 (1.46–2.14)	1.69 (1.38–2.08)
Moderate or heavy	1.85 (1.50–2.27)	1.83 (1.48–2.26)
Lifetime abstainer/status unknown	1 [Reference]	1 [Reference]

**Survey Year**		
2011	1 [Reference]	1 [Reference]
2012	1.00 (0.82–1.23)	0.87 (0.67–1.13)
2013	1.03 (0.84–1.26)	1.07 (0.84–1.37)
2014	1.10 (0.91–1.34)	0.97 (0.75–1.26)
2015	1.32 (1.09–1.60)	1.13 (0.89–1.42)
2016	1.38 (1.15–1.65)	1.31 (1.05–1.63)
2017	1.61 (1.35–1.92)	1.47 (1.18–1.83)
2018	1.84 (1.55–2.19)	1.65 (1.33–2.03)

Abbreviation: —, does not apply.

a Internet-based health care communication use was defined as any use, in the past 12 months, of the following 3 types: communicated with a health care provider by email; filled a prescription on internet; and scheduled a medical appointment on internet.

b Included non-Hispanic ethnicity with one of the following race groups: Native American/Alaska Native only, Asian only, race group not releasable, and multiple race.

c Adults aged ≥65 with private and/or public insurance without Medicare (n = 279) were grouped together with Medicare only.

d The Kessler Psychological Distress Scale (27) consists of 6 questions that ask about feelings of sadness, nervousness, restlessness, worthlessness, hopelessness, and feeling like everything is an effort during the past 30 days. Participants were asked to respond on a Likert scale ranging from none of the time (score = 0) to all of the time (score = 4); a cutoff of 13 was used to dichotomize psychological distress into serious (score ≥13) and not serious (<13).

Unique factors were associated with IBHC in each age group (Table 2). Among survivors aged 18 to 64, we found less IBHC among non-Hispanic Black (aPR = 0.71; 95% CI, 0.58–0.87) and Hispanic survivors (aPR = 0.78; 95% CI, 0.63–0.96) than non-Hispanic White survivors. We found less IBHC also among survivors without private insurance coverage (aPR = 0.63 [95% CI, 0.53–0.73] among the uninsured and aPR = 0.64 [95% CI, 0.52–0.79] among those with public insurance), and among survivors who had received a diagnosis 11 years or more before the survey (aPR = 0.78; 95% CI, 0.69–0.88), compared with survivors who had received a diagnosis 2 years or less before the survey. Among survivors aged 65 or older, unique factors significantly associated with less IBHC were older age (aPR range, 0.36–0.78, all 95% CIs <1), not married (aPR = 0.73; 95% CI, 0.65–0.81), non–US born (aPR = 0.70; 95% CI, 0.54–0.90), and being a former smoker (aPR =1.14; 95% CI, 1.02–1.28).

Patterns of factors associated with the 3 IBHC types were similar to those found in any IBHC use for younger and older cancer survivors (data not shown).

The increasing trend and the patterns in the associated factors of IBHC among participants without a cancer history (data not shown) were similar to the trend and patterns of cancer survivors, although the prevalence of IBHC was approximately 5% to 12% lower than the prevalence among cancer survivors. Among participants without a cancer history, the prevalence of any IBHC use increased from 12.8% in 2011 to 28.3% in 2018 among those aged 18 to 64 and from 8.9% to 20.5% among those aged 65 years or older (both *P* for trend < .001).

## Discussion

Using a large nationally representative sample, we depicted the increasing trend of internet use in communicating with the health care team among cancer survivors from 2011 through 2018. We estimated that in 2018, 40.2% of cancer survivors aged 18 to 64 and 22.6% of cancer survivors aged 65 or older had used IBHC for filling prescriptions, scheduling appointments, or communicating with health care providers. A large proportion of cancer survivors had not used these simple asynchronous forms of telehealth, and, thus, they may be less likely to use more complicated telehealth services, such as video visits. With more than 16.9 million cancer survivors today (19) and the acceleration of telehealth (3,4,16), a pressing need exists to identify and reduce barriers to internet use in patient–provider communication.

We also found unique and common factors associated with disparities in IBHC use among younger and older cancer survivors. Most notably, younger survivors who were non-Hispanic Black or Hispanic or lacked private insurance coverage had significantly lower IBHC. This finding may reflect the general lack of health care access among Black and Hispanic populations compared with their White counterparts and discrepancies in reimbursement policies for telehealth by insurance types (2). Among survivors aged 65 or older, older age was a strong factor associated with lower IBHC. In both age groups, survivors with a high school diploma or less were less likely than survivors with more than a high school diploma to use IBHC. Our findings are consistent with older studies that used data from NHIS (21–23) and studies that used another national population-based survey, the Health Information National Trends Survey (HINTS) (8,9,17,18,20,25). In these studies, younger age, being non-Hispanic White, and higher education were generally found to be associated with higher levels of health-related internet use, although the term was more broadly defined, a use of health information technology and health-seeking behaviors, than the term used in our study.

The concept of the digital divide in the use of health technology — by age and socioeconomic status — is not new, and reasons for the observed disparities are multifaceted, including technology literacy, health literacy, inequitable internet access, and inequitable health care access (2,8,9,21,25,28,29). Despite the growing use of IBHC found in our study and in earlier studies (8,17,21,25), our analysis of nationally representative samples across an 8-year time span confirmed and highlighted the persistence of such sociodemographic disparities in IBHC. The IBHC types analyzed in our study are some of the basic components of telehealth. They are now a ubiquitous component of patient portals in the health care delivery system (6) and may serve as a prerequisite for adopting more complicated technologies. A recent study found that previous experience with online communication with health care providers predicted patient portal use (7).

In addition to sociodemographic disparities, our study revealed disparities in IBHC by health status and health behavior patterns. We found increased IBHC among cancer survivors with comorbid conditions, likely related to their greater health care needs compared with survivors without comorbid conditions. Frequent interaction with health care providers and medical specialists may also increase the likelihood of IBHC use (23). Our result is consistent with the findings from a previous study based on HINTS showing higher online health care provider communication among adults with a cancer history than without a cancer history and among those with poor health status (18). However, time since cancer diagnosis, which may be a surrogate of cancer care needs, was not associated with IBHC among survivors in our study in either age group, except among younger survivors with a cancer diagnosis 11 or more years ago. An earlier study using HINTS data also found that time since cancer diagnosis was not associated with using internet for emailing physicians, buying medicine online, or participating in support groups (17). These nonsignificant findings suggest time since diagnosis may be less relevant to IBHC among cancer survivors. Alternatively, time since diagnosis may not be a precise surrogate for cancer care needs. Moreover, survivors may differ in their health care needs and prognosis according to the type of cancer they have, which can in turn affect their IBHC use. However, our analysis did not address these differences because of small sample sizes.

Although telehealth technologies have long been used to support health behavior counseling and mental health counseling among cancer survivors (10–14), we found higher levels of IBHC use only among alcohol drinkers, not survivors with serious psychological distress or survivors who were current smokers. At the same time, survivors with (vs without) serious psychological distress and survivors who currently (vs never) smoked had significantly lower IBHC use. For cancer survivors, being able to use IBHC is critical in the context of rapid expansion of telehealth along the cancer continuum for health and health behavior promotion. The use of health communication technology may help with physical and mental health wellness and knowledge empowerment among cancer survivors (10–15,30). The gaps identified in our study — low IBHC use among survivors with serious psychological distress and survivors who currently smoke — suggest opportunities for increased awareness and use of IBHC. By extension, increases in IBHC may increase adoption of telehealth tools (eg, mobile health apps, patient portals, video conference platforms), wellness-oriented and clinical services in psychosocial care, and smoking cessation services among cancer survivors.

Taken together, our findings point to sociodemographic and health behavior groups that can benefit from targeted policies and programs aimed at increasing IBHC among cancer survivors. In addition, intervention strategies may need to be tailored for younger and older cancer survivors to address the unique barriers faced by these 2 age groups. The results also call for the use of patient-centered technologies, such as more user-friendly interfaces for the older population, to facilitate effective use of telehealth among underserved groups.

In response to the COVID-19 pandemic, the delivery of telehealth services has changed dramatically (4). For example, before the COVID-19 pandemic, providers were licensed only to provide telehealth services within a state, and Medicare generally reimbursed a limited number of telehealth services for beneficiaries in designated rural areas (2). The Centers for Medicare & Medicaid Services expanded telehealth benefits to include wider ranges of synchronous and asynchronous encounters, added more reimbursement coverage, and relaxed restrictions on the provider types, residence restrictions, and new patient status (4). As of March 31, 2021, most US states and territories had waivers on licensure requirements and renewals for telehealth (31). Such changes may help ameliorate the disparities our study found in insurance types and geographic regions. However, these changes, which removed policy and payment barriers to telehealth during the pandemic, may not all be permanent. Nevertheless, telehealth changes during the pandemic are likely to affect its future use. The baseline information and the disparity patterns about IBHC before the COVID-19 pandemic provided by our study can inform ongoing evaluations of IBHC and other telehealth services use in the future.

Our study has limitations. First, telehealth encompasses a wide range of services but in this study, we were only able to study the basic types of IBHC measured by 3 questions available consistently over time in the survey. Second, self-reported responses may introduce recall bias and social desirability bias. Third, we were not able to assess disease severity and other clinical factors because of a lack of information. Fourth, the cross-sectional data limited our ability to establish causal relationships between survivor characteristics and disparities in IBHC. Fifth, although racial/ethnic minority cancer survivors were oversampled in NHIS, their relatively small number may limit the power in detecting differences by race/ethnicity. In addition, we excluded 872 individuals (4.4%) with missing responses to exposure variables, and these measures may not have been missing at random. Nonetheless, this was a small percentage of our overall sample and was unlikely to affect our findings. A strength of the study was the use of nationally representative samples from high-quality surveys. Another strength was the ability to examine the long-term trends in the use of 3 IBHC types that require interaction with health care teams; this information provides baseline data for evaluating ongoing telehealth use. Finally, our stratified analyses by younger and older cancer survivors offered valuable findings to address their unique barriers in using IBHC.

With expansion of telehealth during the COVID-19 pandemic, our study, by illustrating the prevalence and associated factors of IBHC, provides a national baseline for future research assessing evolution of health technology use among cancer survivors. The identified risk factors can guide targeted efforts to address disparities in using common IBHC and more advanced telehealth technologies among cancer survivors.
